# Mediatorless Impedance Studies with Titanium Dioxide Conjugated Gold Nanoparticles for Hydrogen Peroxide Detection

**DOI:** 10.3390/bios7030038

**Published:** 2017-09-18

**Authors:** Nur Hamidah Abdul Halim, Yook Heng Lee, Radha Swathe Priya Malon Marugan, Uda Hashim

**Affiliations:** 1 School of Chemical Sciences and Food Technology, Faculty of Science and Technology, Universiti Kebangsaan Malaysia, Bangi 43600 UKM, Selangor, Malaysia; leeyookheng@yahoo.co.uk (Y.H.L.); radhaswathepriya@gmail.com (R.S.P.M.M.); 2 Institute of Nano Electronic Engineering, Universiti Malaysia Perlis, Kangar 01000, Perlis, Malaysia; uda@unimap.edu.my

**Keywords:** impedimetric biosensor, mediatorless, direct electron transfer, aminated titanium dioxide, hemoglobin

## Abstract

An impedimetric-based biosensor constructed using gold nanoparticles (AuNP) entrapped within titanium dioxide (TiO_2_) particles for hydrogen peroxide (H_2_O_2_) detection is the main feature of this research. The matrix of the biosensor employed the surface of TiO_2_, which was previously modified with an amine terminal group using 3-Aminopropyltriethoxysilane (APTS) at a low temperature to create a ready to immobilise surface for the biosensor application. Hemoglobin (Hb), which exhibits peroxidase-like activity, was used as the bioreceptor in the biosensor to detect H_2_O_2_ in solution. The analysis was carried out using an alternative impedance method, in which the biosensor exhibited a wide linear range response between 1 × 10^−4^ M and 1.5 × 10^−2^ M and a limit of detection (LOD) of 1 × 10^−5^ M without a redox mediator.

## 1. Introduction

Hydrogen peroxide, H_2_O_2_, is commonly used as a food additive and preservative in food processing, such as in stored milk before cheese processing [1]. In the USA, it is classified to be unsafe if the H_2_O_2_ content is higher than 0.05% relative to the milk weight [2] or more than 14.6 μM in milk samples if according to the FDA [3]. Therefore, the development of a biosensor that can detect H_2_O_2_ within a wide linear range response is of great significance for clinical, pharmaceutical, biochemical, environmental, and food analysis [4].

TiO_2_ is a multi-functional inorganic material that exhibits non-toxic properties, and is chemically inert and thermally stable when enhanced with other metals and semiconducting materials. Furthermore, high catalytic activities can be achieved using TiO_2_ with a large surface area in a dedicated synthesis method [5]. On the other hand, gold nanoparticles (AuNPs) are one of the noble metals, besides palladium and platinum, that have been well studied as an immobilization platform in biosensors due to their high conductivity and electro catalytic behavior [6]. AuNPs also offer a higher surface area compared to flat surface gold, thus allowing greater protein loading, and consequently, a more sensitive biosensor [6]. However, the aggregation between gold and the substrate material imposes an issue [7]. Thus, the stabilized citrate capped reduction method was proposed to prevent this issue [8].

It is noteworthy that the synthesis of a TiO_2_ sphere is complicated and difficult to control as the precursor of titanium (Ti) such as titanium isopropoxide (TTIP) is highly reactive in nature. On the other hand, gold colloids tend to agglomerate in alcoholic solution, which makes it harder to segregate it completely. In other words, an Au/TiO_2_ nanocomposite is prone to rapid agglomeration and inhomogeneous particle distribution [9]. Although the synthesis of a gold core with a thin coating of TiO_2_ has been previously reported by [10], the nanocomposite has only been applied for photocatalytic applications. Furthermore, surface layer modification with an amine functional group on the titanium surface will lead towards better biomolecule immobilization. Although the composite of Ti and Au for biosensor applications has been reported in numerous publications, the construction of an impedimetric biosensor using both an Au/TiO_2_ nanocomposite and enzyme has not been explored. 

Throughout this time, the impedance method has been implemented as the support for biosensor analysis, also known to be a non-destructive characterisation [11]. Besides, the impedimetric methods in measuring an electrochemical biosensor have been applied to detect glucose content [12] and also in bacteria detection [13]. For the latter method, the impedance can be measured because of the metabolites coming from the growth of the bacteria itself. One of the advantages of this method is that it does not require a label especially for the DNA sensor. The impedance value is done by measuring the impedance change due to the target binding on the biorecepetor (either antibody or nucleic acid) that has been immobilised on the electrode surface. 

However, this method is limited to detecting small analytes and metabolites and exposure to non-specific adsorption [14]. The application focuses on the detection of antibodies [15], bacteria [13], cholestrol [11,12], cancer cells [13,16], and DNA [17]. This EIS is mainly used for the surface characterisation of chemical sensors and biosensors and has been practised for a long time and is efficient for label-free detection, especially for a DNA biosensor [11,18]. Furthermore, in most impedance analysis redox couples such as ferricyanide/ferrocyanide, an electron acceptor for a heme containing enzyme [19] is often introduced but it may cause complications or interactions between the target molecule and probe surface [20]. 

In this study, AuNPs were entrapped within aminated TiO_2_ to ease the immobilization of the biomolecule in biosensor development. In addition, Hb was covalently attached onto the modified TiO_2_ surface. The AuNPs entrapped within TiO_2_ were modified, in which an amide bond was formed between the carboxyl end terminal of Hb and the amine terminal from TiAu-APTS. The TiAu-APTS is believed to provide catalytic properties towards the enzymatic sensor. It is also noteworthy that the impedimetric biosensor was developed without the usage of any redox mediators. In previous work, this conjugated TiAu-APTS was studied using the differential pulse voltammetry method with an acceptable linear range response towards the H_2_O_2_ concentration.

## 2. Materials and Methods

The precursor reagent for titanium dioxide particles was titanium isopropoxide (TTIP, 95%). Absolute ethanol (99.5%) was used as the main solvent. Ammonia (25%) was used as the catalyst in this synthesis. 3-Aminopropyltriethoxysilane (APTS, 99%), lyophilized human hemoglobin, gold chloride trihydrate (HAuCl_4_, 49%), *N*-(3-Dimethylaminopropyl)-*N*′-ethylcarbodiimide hydrochloride (EDC), *N*-hydroxysuccinimide (NHS, 98%), and 2-(*N*-Morpholino)ethanesulfonic acid (MES) hydrate buffer were purchased from Sigma Aldrich. Trisodium citrate dihydrate that was purchased from Systerm was used for the preparation of the Au colloid. All the chemicals purchased were of analytical grade and used without further purification. The phosphate buffer solution was prepared by mixing disodium phosphate (Na_2_HPO_4_) and sodium dihydrogen phosphate (NaH_2_PO_4_). Deionized water (DIW, 18 MΩcm) was used throughout this experiment.

TiAu-APTS was prepared using a slight modification of a method reported before [21,22,23], with TiO_2_ conjugated with the Au colloid and later functionalized with APTS (Additional Supplementary Data). The morphology of TiAu-APTS was then observed under a Field Emission Electron Microscope (FESEM) and Transmission Electron Microscope (TEM). 

**Figure 1:**
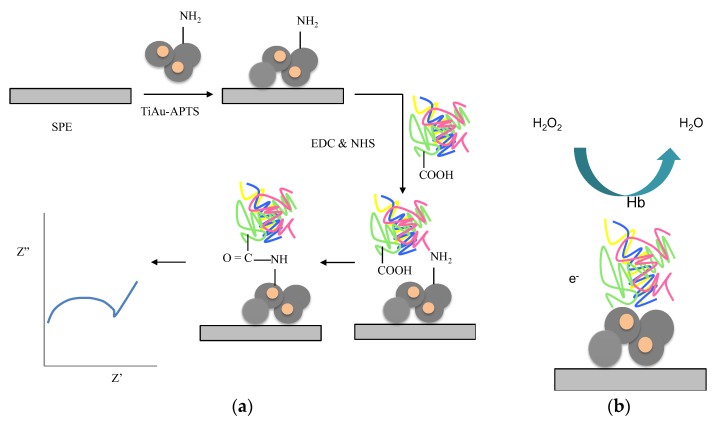
(**a**) Fabrication steps and (**b**) mechanism of the Hb/TiAu-APTS/SPE impedance biosensor using the EDC-NHS route for Hb immobilization on the modified electrode.

The fabrication steps of the electrode Hb/TiAu-APTS/SPE are illustrated in Figure 1a. A TiAu-APTS colloid of 15 μL was dropped onto the SPE and left to dry at room temperature. Hb was first activated separately using EDC (0.02 M) and NHS (0.004 M) in MES buffer (pH 6.85) at room temperature. Subsequently, the prior prepared TiAu-APTS/SPE was immersed into the activated Hb in EDC/NHS for nine hours immobilisation. The Hb/TiAu-APTS/SPE was rinsed thoroughly before analysis. All the experiments were carried out using CV and EIS methods in 0.05 M Na PBS (pH 7), unless otherwise stated. 

An AutoLAB potentiostat PGSTAT 12 (Metrohm) was used to perform all the electrochemical measurements. It includes a modified carbon Screen printed electrode (SPE) (Scrint Technology (M) Sdn. Berhad), Pt electrode, and Ag/AgCl (in saturated KCl) electrode as the working, counter, and reference electrodes, respectively. The Cyclic Voltammetry (CV) and Differential Pulse Voltammetry (DPV) were conducted to observe the electrochemical behavior of modified electrodes in both buffer (0.05 M, pH 7.0) and potassium ferricyanide (5 mM) solution. The CV experiments were carried out versus the Ag/AgCl reference electrode at room temperature (25 °C) at a scan rate of 100 mV/s. Electrochemical Impedance Spectroscopy (EIS) measurements were performed at frequencies from 100 kHz to 100 Hz with an amplitude (0.1) to the open circuit potential (OCP) in buffer solution. The impedance results were recorded at a DC potential of ±200 mV and the impedance spectrum was acquired at seven minutes. The calibration curve was produced by preparing 30 SPEs at various concentrations of 1 × 10^−6^ to 1.5 × 10^−2^ M H_2_O_2_ using the Hb/TiAu-APTS/SPE electrode.

## 3. Results and Discussion

### 3.1. Characterization of TiAu-APTS

**Figure 2:**
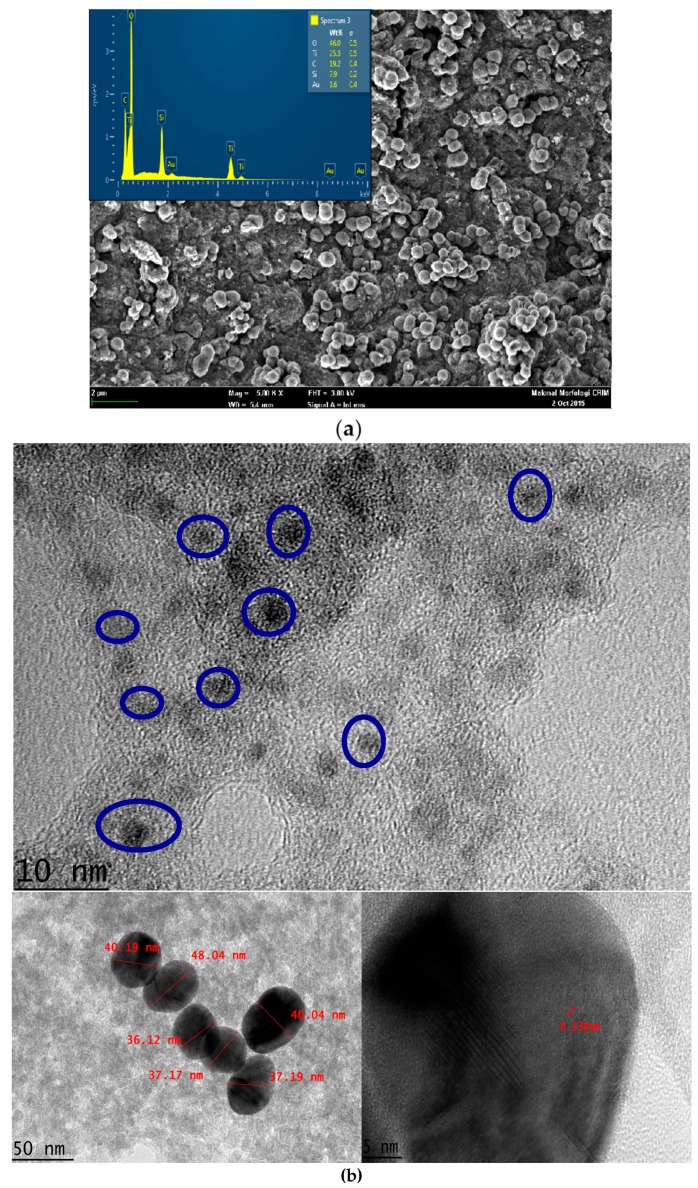
(**a**) TiAu-APTS under SEM with the inset of EDX spectrum and (**b**) Morphology of TiAu-APTS under TEM. The blue circle shows the Au colloid that is embedded with TiO_2_ particles in an amorphous state. The bottom left inset is an Au nano colloid with TiO_2_ particles. The bottom right inset shows the Au nano colloid crystal with d = 0.2368 nm.

The TiAu-APTS on the electrode was characterized using FESEM and TEM, as shown in Figure 2a,b, respectively. It was observed that the TiAu-APTS particles were of a non-uniform shape with submicron sizes of less than 1 μm. The EDX spectrum shows the elements of Ti, SiO, and Au on the surface of TiAu-APTS. The Si was observed due to the silane group derived from APTS during the synthesis. From the TEM observation, it was observed that the Au NPs were trapped within TiO_2_ particles. The faint image of AuNPs is due to the thick particles of the amorphous TiO_2_ blocking transmission of the electron of the AuNP. The TiAu produced AuNPs with a size of 4–5 nm, which is much smaller than the average size of the AuNP colloid of ±38.85 nm before the synthesis took place. The TiO_2_-Au conjugate is proportionately bigger in size with thicker TiO_2_ amorphous particles and reduced Au colloid sizes. The change in the Au size happened due to the addition of ammonia during the synthesis that gradually changes the pH of the solution to become more basic, thus reducing the size of AuNP. The size reduction of AuNP due to the pH solution is in agreement with Brinas et al., 2008 [24].

### 3.2. Characterization of AuNPs/SPE and TiAu-APTS/SPE Electrode 

**Figure 3:**
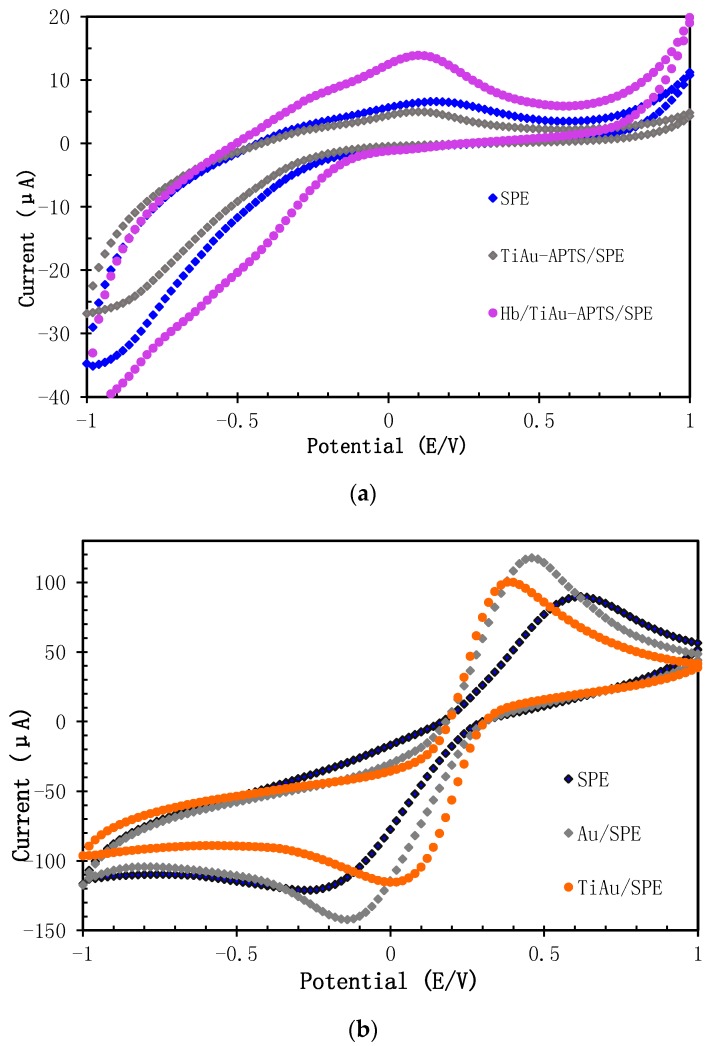
Electrochemical study of different modified SPEs in (**a**) 0.1 M PBS (0.1 M NaCl, pH 7.4) and (**b**) in 5 mM K_3_[Fe(CN)_6_] solution.

First, Na PBS (0.05 M, pH 7.4, 0.075 NaCl) was used to study the characteristics of Hb/TiAu-APTS/SPE. Figure 3a shows the CV of SPE, TiAu-APTS/SPE, and Hb/TiAu-APTS/SPE in Na PBS. It can be observed that the oxidation peak for the SPEs that were immobilised with Hb (Hb/TiAu-APTS/SPE) was higher compared to TiAu-APTS/SPE. This confirmed that Hb was succesfully immobilised on the SPE due to the presence of the reduction peak at approximately −0.3 V, which is in agreeement with [25]. The oxidation peak values were Hb/Au/SPE > Hb/SPE > Hb/TiAu-APTS/SPE. The low oxidation peak value was due to the semiconductive properties of TiAu-APTS in buffer solution and the absence of a redox probe such as ferricyanide ([Fe(CN)_6_]^3−^). The electron transfer rate in Na PBS is also slow as the anodic and cathodic peak separation value (ΔEp) is 450 mV. In comparison, Figure 3b shows the CV of the modified SPEs in a conductive solution containing potassium ferricyanide (K_3_[Fe(CN)_6_]) as the redox probe. AuNPs are well known for their excellence conductivity, and are introduced to enhance the semiconducting properties of TiO_2_. This AuNP embedded inside the titanium oxide demonstrated better electron transfer as TiAu-APTS/SPE resulted in a better electron transfer rate (ΔEp of 279 mV) compared to the Au/SPE electrode (ΔEp of 559.6 mV).

**Figure 4:**
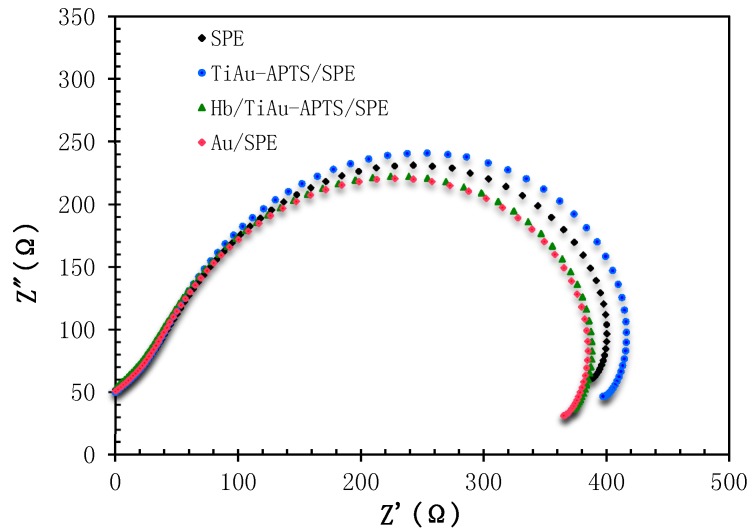
The Nyquist plot of different modified SPEs in PBS (0.1 M NaCl, pH 7.4).

Figure 4 shows a Nyquist plot for the surface study of SPE, Au/SPE, TiAu-APTS/SPE, and Hb/TiAu-APTS/SPE electrodes in Na PBS. It can be observed that bare SPE has a higher electron charge transfer resistance (R_CT_) value compared to Au/SPE and Hb/TiAu-APTS/SPE, but a lower R_CT_ value than TiAu-APTS/SPE. The Z′ value that is derived from the semi-circle axis also represented by R_CT_ represents the kinetic electron transfer value of the interface electrode [11]. The lowest impedance value (369 Ω) was obtained from Au/SPE due to the highly conductive property of gold that lowers the resistance on the electrode surface. On the other hand, TiAu-APTS/SPE has the highest impedance value (401 Ω) due to the poor electrical conductivity of TiAu-APTS/SPE. However, when Hb was immobilized on the TiAu-APTS/SPE (Hb/TiAu-APTS/SPE), it gives a lower impedance value (372 Ω) than TiAu-APTS/SPE because the immobilized protein has good conformation and is capable of maintaining its natural activity in a suitable condition [11]. The low impedance value in Hb/TiAu-APTS/SPE reflects the low resistivity and better current penetration of the electrode in mediatorless buffer solution. 

In impedance analysis, high impedance values are obtained when electrodes with a similar charge repel each other in the interface between the bulk solution and surface of the electrode. Unlike amperometric analysis, the mechanism of the redox process in impedance analysis is still unclear. Hence, the impedance value of a conductive material depends on the optimum size and quantity that can either reduce or increase the impedance due to the repelling charge effect. When similar charges are built up on the surface of the electrode, a diffusion process can develop for charge transfer resistance (R_CT_), and the process of electrons moving to the electrode surface becomes slower, thus increasing the R_CT_ values [18]. It is important to have an optimum Hb immobilized on the modified electrode surface that results in enough electron transfer and avoids creating a repelling charge effect that increases the R_CT_ value. 

### 3.3. Performances of Biosensor 

**Figure 5:**
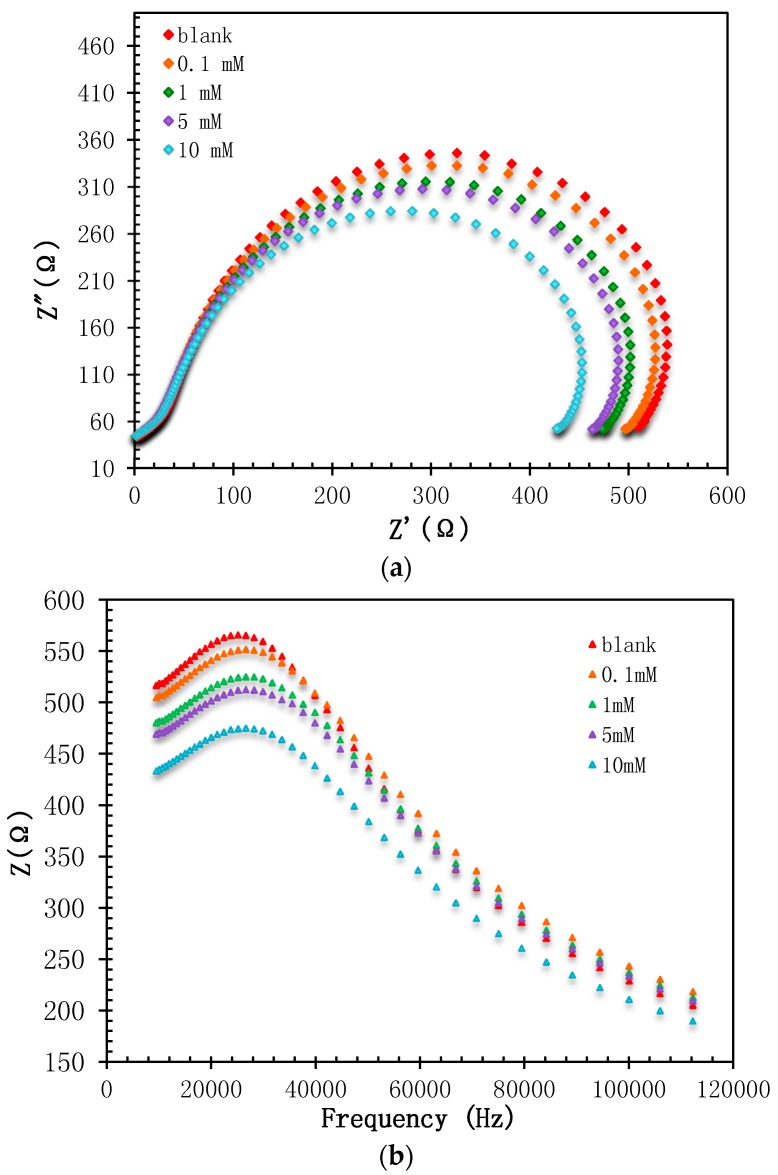
The (**a**) Nyquist and (**b**) Bode modulus plot for the Hb/TiAu-APTS/SPE biosensor at different H_2_O_2_ concentrations.

H_2_O_2_ concentrations in the range between 5 × 10^−1^ M and 5 × 10^−10^ M were used to determine the preliminary dynamic range of the Hb/TiAu-APTS/SPE biosensor. Figure 5a shows the Nyquist plot for different H_2_O_2_ concentrations. The Nyquist plot consists of two significant parts that are a semicircle and linear part that are represented by an electrode interface on the surface and electrode diffusion layer, respectively. The value represents the kinetic electron transfer of the redox probe at the electrode interface [11]. The R_CT_ value was lower at higher concentrations of H_2_O_2_ and this finding was supported by [26,27]. 

Lin et al. [27] reported a linear range for H_2_O_2_ detection between 4 × 10^−5^ and 1 × 10^−4^ M with a low LOD of 2 × 10^−6^ M, where the response time was 30 to 40 min. However, for this Hb/TiAu-APTS/SPE biosensor, the response time was 3 min. The low R_CT_ is believed to occur because of the production of an H^+^ proton in the measurement solution [28] whenever Hb was reacted with a higher concentration of H_2_O_2_. In the absence of electron mediator [Fe(CN)_6_]^3−/4−^, the performance of the Hb/TiAu-APTS biosensor was in agreement with the mechanism reported [29], as depicted in Figure 1b. The impedance R_CT_ value may increase or decrease depending on the difference in the charge on the surface of the electrodes and solution. At low H_2_O_2_ concentrations, the R_CT_ value was at the highest because electron movement was blocked. On the other hand, at a high H_2_O_2_ concentration, a low impedance value was recorded, which indicated that electrons passing through the modified electrode were not blocked. This result was in agreement with the trends reported in [30], where the R_CT_ value decreased with increasing H_2_O_2_ concentrations. The resistivity of the film becomes greater whenever H_2_O_2_ is at a low concentration. 

The bode modulus was plotted as shown in Figure 5b to observe the impedance change, Z or constant phase element, and CPE against the frequency. The plot was important because impedance is not solely determined based on the R_CT_ value, but also on the frequency position during the reaction. From the plot, it can be observed that the frequency was recorded from 26 to 28 kHz. 

**Figure 6:**
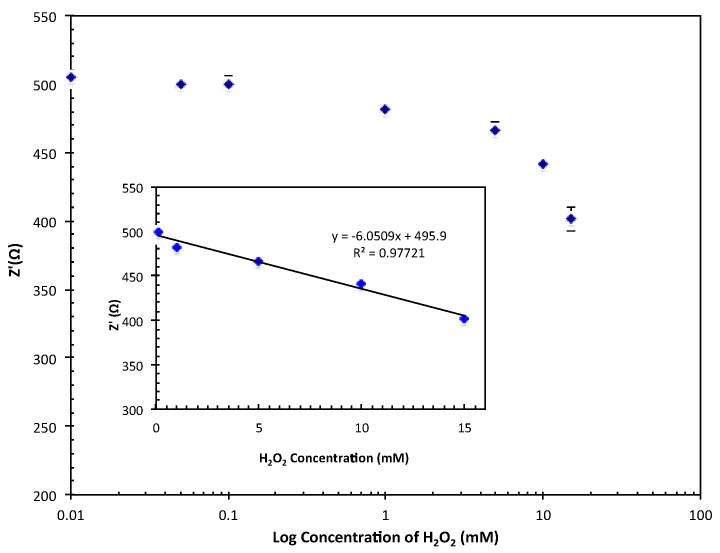
The response linear range of Hb/TiAu-APTS/SPE biosensors towards various H_2_O_2_ concentrations.

Based on Figure 6, the linear range for the Hb/TiAu-APTS/SPE biosensor was recorded between 1 × 10^−4^ and 1.5 × 10^−2^ M with a low LOD of 1 × 10^−5^ M. The linear range was narrower than reported in [7] between 1 × 10^−5^ and 22.3 × 10^−3^ M. Regardless, this Hb/TiAu-APTS/SPE biosensor gave a better response than the enzymeless TiO_2_-Ag biosensor reported by [7], which only had an LOD of 5 × 10^−4^ M H_2_O_2_. Overall, the performance of this Hb/TiAu-APTS/SPE biosensor was less sensitive compared to the amperometry method due to its narrow linear range, but it can be used as an alternative method to qualitatively support an amperometry biosensor on the electrode surface considering that the diffusion process in kinetic and mass transfer is limited. The measurement in the absence of redox mediator [Fe(CN)_6_]^3−/4−^ produced a narrow linear range response, yet still managed to detect the analyte. 

In addition, an interference study was conducted using common interferences from electroactive species such as ascorbic acid and glucose at three different ratios to observe the selectivity of the biosensor, and no significant interferences were observed in Table 1 as small changes in R_CT_ within experimental errors were obtained when the interferants were introduced. However, electroactive species such as ascorbate act as molecular oxygen activators producing free radicals [31] if combined with radical H_2_O_2_, creating interference towards the response of a biosensor at a much higher concentration of H_2_O_2_. 

From the Nyquist plot, the R_CT_ value on the *x*-axis and *y*-axis refers to the resistance and capacitance value, respectively. In comparison to the DPV method, the impedance can be regarded as the reciprocal of the current value in the electrochemical measurement. As in the electrochemical measurement, the reactions of different analytes occur at a specific potential. However, in impedimetric measurements, the frequency is also important to qualitatively determine the response of different analytes for developing biosensors. However, the drawback of the impedance analysis method using a single frequency is its slow response compared to the amperometry method [12].

**Figure 7:**
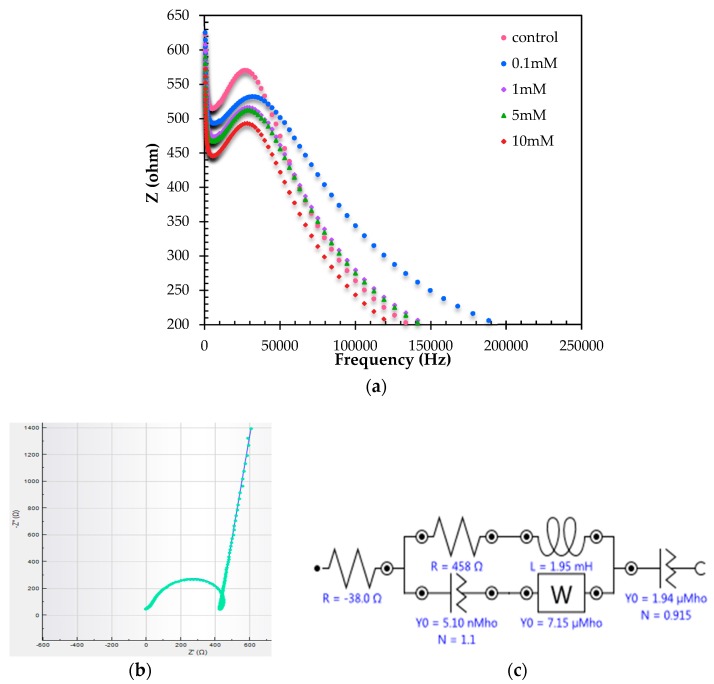
The (**a**) Bode modulus plot for the real milk sample at 33.5 kHz and (**b**) Nyquist plot of the real and (**c**) fitted equivalent circuit to represent the impedance biosensor of the Hb/TiAu-APTS/SPE biosensor with χ^2^ ≤ 0.1.

A commercial milk sample was used to validate the accuracy of this Hb/TiAu-APTS/SPE biosensor method with the standard method. When Hb was exposed to a real sample of diluted milk in the ratio of 1:11, the frequency was recorded at 33.5 kHz, but the frequency shifted to 26.6 kHz in buffer solution, as shown in Figure 7a. Figure 7b shows the equivalent circuit that represents the Hb/TiAu-APTS/SPE biosensor. The circuit is constructed from a resistor, R_s_, which is connected to a parallel resistor and inductor, L, with CPE and Warburg impedance, and finally with another CPE element. The inductance may be derived from the magnetic effect of the Fe^3+^ ion inside Hb to form an inductor in the system. However, there might be a possibility that the inductor is not a real inductor, but from a resistor and capacitor. Table 2 shows the recovery results for the commercial milk sample at four different H_2_O_2_ concentrations. The recovery of this biosensor within the range of 90 to 114% at four different H_2_O_2_ concentrations is acceptable for the recovery of milk analysis reported by Dong et al., which was reported to be between 94.3% and 119% at LOD 3.3 μM [2].

## 4. Conclusions

This Hb/TiAu-APTS/SPE biosensor with impedance detection demonstrated a satisfactory linear range response for H_2_O_2_ detection with a good resistance to interference. The mediatorless impedance biosensor with an LOD of 1 × 10^−5^ M and a reproducibility of 5% (*n* = 3) shows enhancement in the ability of H_2_O_2_ to be detected in the milk sample within the range of the safety limit of 14.6 μM according to the FDA. Even though the demonstrated process was tedious, it opens up the challenge to explore impedimetric areas in biosensing. This biosensor also demonstrated the ability of Hb to be immobilised solely on the TiAu-APTS surface, and the change in the biomolecule charge in Hb during exposure to analyte was thus successfully observed through this impedance study. 

## Figures and Tables

**Table 1:** Interference study on the Hb/TiAu-APTS/SPE biosensor towards glucose and ascorbic acid with 1 mM H_2_O_2_ at different ratios (*n* = 3).

Ratio of Interference to Analyte	Glucose Impedance Value (R_CT_)	% Change	Ascorbic Acid Impedance Value (R_CT_)	% Change
High (10:1)	433.67	−1.66	408.00	−4.90
Medium (1:1)	419.67	−2.18	414.67	−3.34
Low (0.1:1)	417.33	−2.72	438.33	2.18

**Table 2:** Recovery of the milk sample for the Hb/TiAu-APTS biosensor in H_2_O_2_ detection (*n* = 3) with RSD = 6%.

Added H_2_O_2_ (mM)	Found in Milk Sample	Recovery %
1	1.14	114
5	4.94	98
10	9.02	90
15	15.63	104

## Supplementary Materials

The following are available online at www.mdpi.com/2079-6374/7/3/38/s1.
