# Supplementary material for: Mediatorless Impedance Studies with Titanium Dioxide Conjugated Gold Nanoparticles for Hydrogen Peroxide Detection
Source: Biosensors (Basel). 2017 Sep 18;7(3):38. doi: 10.3390/bios7030038 (PMC5618044; doi:10.3390/bios7030038)
Supplement: Supplementary file 1 [file biosensors-07-00038-s001.pdf]

Additional Supplementary Data

Synthesis of Titanium Dioxide and TiAu-APTS

First, AuNPs colloid was synthesized using typical citrate reduction method from [1] in which trisodium citrate solution was added into boiled $HAuCl_4$ solution. The resulting red wine solution indicated the formation of AuNPs and used for the preparation of TiAu. On the other hand, Ti-APTS was prepared using ammonia based on a method reported before [2] and later modified with APTS.

The conjugation of TiO_2 with Au colloid, expressed as TiAu was then synthesized using a slight modification of a method reported before [3-5]. First, the prior prepared AuNPs colloid (4 mL) was mixed and stirred with ammonia (1 mL) for 10 minutes. At the same time, TTIP (1 mL) was added into a mixture of ethanol (10 mL) and DIW in a separate beaker. The mixture was then added into the prepared Au-ammonia solution and left stirred at room temperature for 24 hours. Finally, the TiAu colloid was separated via centrifugation and washed several times with ethanol and DIW to obtain TiAu particles.

The surface of the TiAu particles was then modified with amine by immersing it in APTS solution containing ethanol and stirring it overnight. The TiAu-APTS was then centrifuged at 4000 rpm for 25 min and washed several times before dispersing it in 4 mL of DIW. The resulting colloid was stored away from light at 4 °C.

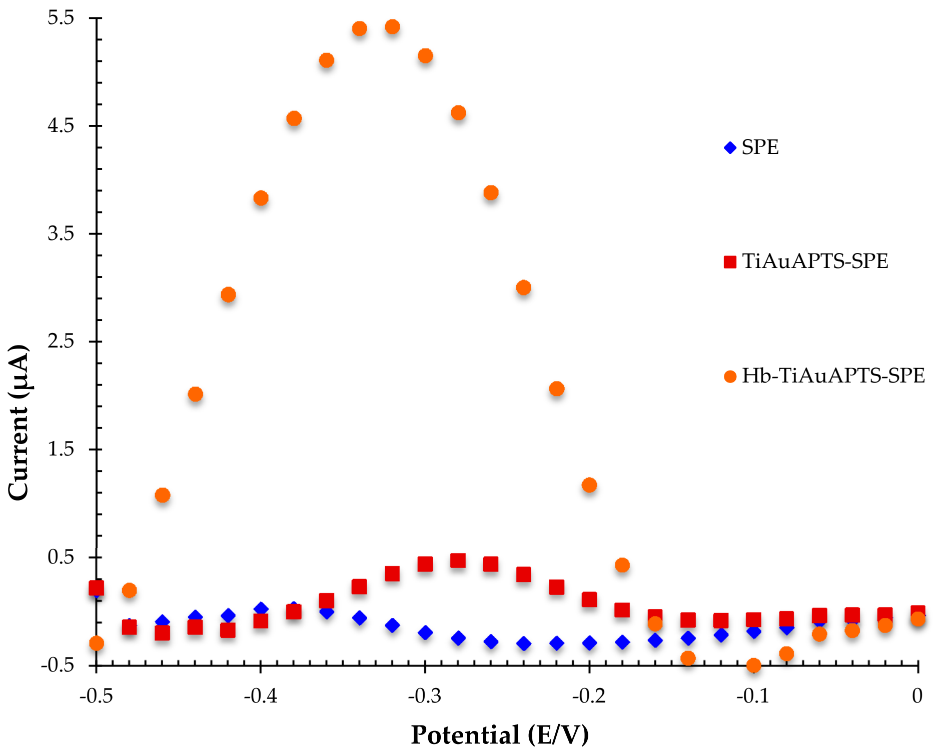

Figure 1 DPV of modified electrode of TiAuAPTS-SPE before and after Hb immobilization in PBS solution pH 7.

Table 2 Analysis of equivalent circuit of [R([CRL][QW])R(QQ)] of the value of R_s, R_{CT}, W and CPE for the impedance biosensor Hb/TiAu-APTS/SPE with χ^2= <0.1.

H_2O_2 Concentration	R_s (Ω)	R_{CT} (Ω)	W (μMho)	CPE (nMho)	L
control	-11.8	513	8.54	4.54	2.43
0.1 mM	-13.5	511	8.09	4.33	2.25
1 mM	-13.6	484	8.16	4.64	2.18
5 mM	-14.2	476	8.17	4.72	2.14
10 mM	-14.4	442	8.32	5.82	2.24

Table 3 The comparison of Hb based TiAu-APTS and Ti-APTS biosensor performances towards H_2O_2 detection.

Biosensor	Linear Range	Limit of Detection	Stability	Detection Method	Redox Probe
Hb/Ti-APTS/Au/SPE	1×10^{-4} - 1×10^{-2} M	1×10^{-4} M	-	Voltammetry	$[Fe(CN)_6]^{-3}$
Hb/TiAu-APTS/SPE	5×10^{-5} - 5×10^{-2} M	5×10^{-6} M	70 % (30 days)	Voltammetry	$[Fe(CN)_6]^{-3}$
Hb/TiAu-APTS/SPE	1×10^{-4} -1.5×10^{-2} M	1×10^{-5} M	90 % (60 days)	Impedimetry	None

[1] Liu J., Lu Y., *Nat Protocol*, 2006, 1 (1), 246-252.

[2] Mine E., Hirose M., Nagao D., Kobayashi Y., & Konno M., *Journal of Colloid and Interface Science*, 2005, **291**(1), 162–168.

[3] Wu Z., Jinglun Liang, Xiaohui Ji, and Wensheng Yang, *Colloids and Surfaces A: Physicochemical and Engineering Aspects*, 2011, 392220-224.

[4] Liu S., Han M.Y., *Advanced Functional Materials*, 2005, **15** (6), 961-967.

[5] Seh, Z. W., & Liu, S., *The Royal Society of Chemistry*, 2011 1–7.

[6] Pei, S., Qu, S., & Zhang, Y. (2010). Direct Electrochemistry and Electrocatalysis of Hemoglobin at Mesoporous Carbon Modified Electrode. *Sensors*, *10*(2), 1279–1290.
